# Real-time and resource-efficient banana bunch detection and localization with YOLO-BRFB on edge devices

**DOI:** 10.3389/fpls.2025.1650012

**Published:** 2025-08-21

**Authors:** Shuo Wang, Lijiao Wei, Danran Zhang, Ling Chen, Weihua Huang, Dongjie Du, Kangmin Lin, Zhenhui Zheng, Jieli Duan

**Affiliations:** ^1^ Agricultural Machinery Research Institute, Chinese Academy of Tropical Agricultural Sciences, Guangdong, China; ^2^ Key Laboratory of Agricultural Equipment for Tropical Crops, Ministry of Agriculture and Rural Affairs, Guangdong, China; ^3^ Institute of Agricultural Machinery, Chinese Academy of Tropical Agricultural Sciences, Guangdong, China; ^4^ College of Engineering, South China Agricultural University, Guangdong, China; ^5^ College of Mathematics and Informatics, South China Agricultural University, Guangdong, China

**Keywords:** machine vision, detection and localization, banana bunches, lightweight model, edge computing

## Abstract

Reliable detection and spatial localization of banana bunches are essential prerequisites for the development of autonomous harvesting technologies. Current methods face challenges in achieving high detection accuracy and efficient deployment due to their structural complexity and significant computational demands. This study proposes YOLO-BRFB, a lightweight and precise system designed for detection and 3D localization of bananas in orchard environments. First, the YOLOv8 framework is improved by integrating the BasicRFB module, enhancing feature extraction for small targets and cluttered backgrounds while reducing model complexity. Then, a binocular vision system is used for localization, estimating 3D spatial coordinates with high accuracy and ensuring robust performance under diverse lighting and occlusion conditions. Finally, the system is optimized for edge-device deployment, achieving real-time processing with minimal computational resources. Experimental results demonstrate that YOLO-BRFB achieves a precision of 0.957, recall of 0.922, mAP of 0.961, and F1-score of 0.939, surpassing YOLOv8 in both recall and mAP. The average positioning error of the system along the X-axis is 12.33 mm, the average positioning error along the Y-axis is 11.11 mm, and the average positioning error along the Z-axis is 16.33 mm. The system has an inference time of 8.6 milliseconds on an Nvidia Orin NX with a GPU memory requirement of 1.7 GB. This study is among the first to focus on a lightweight approach optimized for deployment on edge computing devices. These results highlight the practical applicability of YOLO-BRFB in real-world agricultural scenarios, providing a cost-effective solution for precision harvesting.

## 1 Introduction

Banana is a vital dual-purpose crop for food and fodder in tropical regions, with a global harvested area of approximately 5 million hectares (Zheng et al., 2024a). As the most widely exported fresh fruit worldwide, the modernization of the banana industry plays an irreplaceable role in addressing global hunger and promoting socioeconomic development (Thai et al., 2025). Among the challenges to automation in banana production, accurate and efficient localization of banana is a critical bottleneck. However, existing systems for banana detection suffer from low accuracy, slow processing speeds, and high costs, limiting their applicability for widespread deployment. Hence, designing an efficient and compact neural network-based approach for banana fruit detection is essential to address automation challenges in harvesting machinery and to promote the technological advancement of the banana production sector.

Research on fruit recognition technologies has been conducted extensively and can be broadly categorized into traditional machine learning methods and deep learning approaches (Zheng et al., 2021). Traditional image recognition techniques include threshold segmentation (Tong et al., 2024), clustering-based edge detection (Zhang et al., 2022b), and support vector machines (Liu et al., 2018). Wang (2024) applied K-means clustering and the Sobel operator to extract the contours of Rosa roxburghii fruits, achieving an average size recognition error of as low as 1.22%. Wu et al. (2021) enhanced the K-means clustering algorithm by optimizing its centers with a disturbance-factor-modified gray wolf optimization algorithm, resulting in an average fruit recognition rate of 89.2%. Liu et al. (2019) segmented apple images into superpixel units using the simple linear iterative clustering algorithm and classified them into fruit and background categories with SVM, achieving a segmentation accuracy of 92.14%. Wang (2017) processed kiwi images using the Renyi entropy thresholding method, extracted samples with a minimum enclosing matrix algorithm, and modeled them with SVM, yielding a recognition rate of 87.67%. Fu et al. (2019) employed an SVM-based method that combined local binary pattern features and multi-feature fusion techniques for banana detection, achieving an average single-scale detection rate of 89.63% with a processing time of 1.325s. These studies demonstrated effective fruit detection by leveraging hand-crafted features with traditional machine learning methods. However, their robustness in complex orchard environments remains limited, and the reliance on manual feature extraction constrains the performance and efficiency of such methods, hindering their practical applications.

In recent years, deep learning has gradually replaced traditional methods and found applications in smart agricultural production Zheng et al., 2024b; Zhang et al., 2022a; Zheng et al., 2023; Li et al., 2022a; Ganesan et al., 2022; Li et al., 2022b; Bai et al., 2022). The multi-layered structure of convolutional neural networks enables the extraction of high-level feature representations, offering significant advantages in solving object detection problems (Hu et al., 2021). Duan et al. (2018) developed Panicle Net, based on the SegNet fully convolutional network, for offline training. The network segmented sub-images, which were then stitched together, overcoming irregular panicle edges and interferences such as cultivar and environmental factors. It operated at speeds approximately 35 times faster than Panicle-SEG. Xu et al. (2021) used ExG factors and the Otsu algorithm to segment rice images, followed by ResNet50, optimized with the RAdam optimizer, for growth stage recognition, achieving 97.33% accuracy with high network stability and rapid convergence. Fu et al. (2020a) proposed YOLO-banana, a YOLOv4-based detection network, attaining an AP of 99.55% for multi-class banana fruit detection in orchard environments. Duan et al. (2022) enhanced the YOLOv5 algorithm by integrating the CA attention mechanism into its backbone network and creating the C3CA module. This achieved an average precision of 99.29% for banana stalk-base localization. Zhu et al. (2022) used a UNet model augmented with multi-scale atrous convolution to increase receptive fields while preserving detail sensitivity, achieving an average pixel classification accuracy of 97.32% for banana bunch segmentation. Cai et al. (2023) proposed YOLOv7-FM, an improved YOLOv7 algorithm for detecting banana pseudostems under various growth conditions, reporting an AP of 81.45% and an average inference time of 8.0 ms per image. Neupane et al. (2019) employed Fast-RCNN to analyze UAV-collected images for banana plant detection and counting, with accuracies of 96.4%, 85.1%, and 75.8% across three regions. Wang et al. (2023) utilized YOLOv5 to recognize banana peduncles, achieving an AP of 98.034% with an IoU threshold of 0.5. Despite the success of these methods, most rely on high-performance workstations due to their high parameter counts, making them unsuitable for the low-cost, high-efficiency demands of agricultural operations. Additionally, these studies primarily focus on fruit recognition, while further obtaining 3D coordinates is essential for precise localization and automated harvesting.

The primary methods for obtaining 3D coordinates include structured light (Zhou et al., 2024), Time of Flight (Fu et al., 2020b; Wu et al., 2022, 2023; Gené-Mola et al., 2019), and stereo vision (Xiong et al., 2018; Wang et al., 2019). For structured light, Zhou et al. (2024) introduced a structured light-based technique aimed at assessing the weight of banana clusters in orchards and pinpointing the stem centers with precision. However, structured light is highly sensitive to ambient light interference, and its accuracy decreases significantly with increasing distance, making it unsuitable for practical harvesting scenarios in banana orchards. For ToF, Wang et al. (2019b) leveraged time-of-flight (ToF) imaging to map entire apple tree environments, effectively acquiring precise three-dimensional spatial data for apple localization. Yao (2023) proposed a hand-eye calibration method integrating a ToF depth camera, achieving an average positioning error of less than 4 mm. Lai (2023) utilized ToF cameras to acquire depth images and generate point clouds, applying the RANSAC algorithm to compute the size and 3D position of fruits, with an average correct recognition rate of 84.8%. However, ToF cameras generally have low resolution, which hampers subsequent fruit image recognition. Additionally, they are prone to interference from strong reflections and translucent objects; for example, banana leaves could significantly affect their accuracy, rendering them unsuitable for practical harvesting in banana orchards. For stereo vision, Zheng et al. (2024) used a deep learning-based CREstereo matching algorithm to establish a binocular vision system, achieving an average 3D positioning error of 5.99 mm for cherry tomato pedicels. Wang et al. (2019a) proposed a matching and positioning method using a binocular vision system with a window-scaling mechanism, achieving an average recognition accuracy of 96.33% under six different conditions. Compared to the other two methods, stereo vision is less affected by ambient light, exhibits higher robustness, and provides depth information with higher resolution, making it highly advantageous for precise fruit positioning and harvesting in complex banana orchard environments.

Unlike previous studies, this research introduces a novel banana bunches detection model, named YOLO-BRFB, which is based on active dual-infrared stereo vision technology. By incorporating multi-point sampling and filtering optimization, the proposed model enables accurate 3D positioning of targets during fruit harvesting. By enhancing critical system modules, this research presents a fully integrated and cost-effective solution for fruit identification and localization. The primary contributions of the work are outlined as follows:

(1) This study introduces YOLO-BRFB, a banana detection model developed by integrating the BasicRFB module into the YOLO framework.

(2) The research proposes a dual-infrared active stereo vision system for precise 3D localization of banana clusters. This method leverages multi-point sampling and advanced filtering techniques to mitigate the effects of occlusion and environmental noise.

(3) The proposed system was optimized for deployment on edge devices, achieving a balance between high detection performance and resource efficiency.

## 2 Materials and methods

### 2.1 Image collection and preprocessing

**Figure 1:**
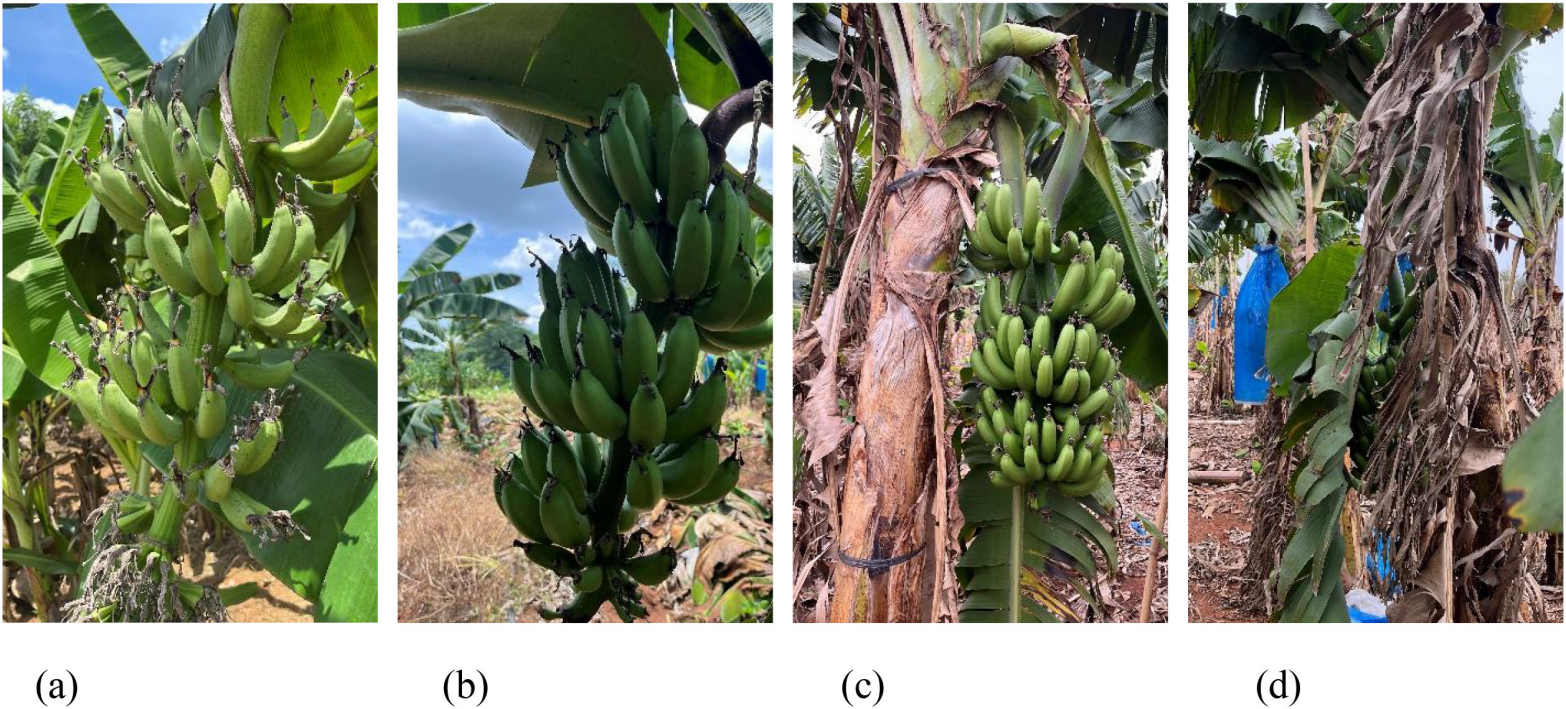
Multiple complex scene images in orchard environment. **(a)** Direct lighting **(b)** Backlighting **(c)** Unocclusion **(d)** Occlusion.

Banana fruit images were gathered from the plantation of the South Subtropical Crops Research Institute (Zhanjiang, Guangdong, China; 21°10′N, 110°16′E) during three time periods: October and November 2023, and June 2024. Each banana plant was individually photographed using handheld devices, including a HUAWEI Mate 60 Pro and an iPhone 13, positioned approximately 70–80 cm from the target fruit. The images, stored in JPG format at a resolution of 4032 × 3024 pixels, were captured under natural lighting conditions. In total, 1500 high-resolution color images were obtained. To enhance the dataset’s diversity, various environmental scenarios such as strong illumination, shadowing, backlight, occlusion, and clear visibility were included. **Figure 1** illustrates representative examples under these conditions.

Dataset quality significantly influences the precision of recognition models and their reliability in real-world deployment scenarios. To ensure clarity and representativeness, the collected banana samples underwent a quality screening process. Images that were excessively blurred, duplicated, or without visible banana fruits were excluded, resulting in a dataset of 1450 valid banana images. The selected images were manually annotated using LabelImg in YOLO file format. During the annotation process, we followed a consistent labeling protocol: (1) Bounding boxes were drawn to tightly enclose the visible banana bunch area, including partial occlusions when at least 60% of the bunch was visible. (2) In cases of overlapping fruit or background clutter, annotators prioritized the primary bunch in focus and excluded indistinct or heavily blurred bunches. To ensure effective model training and validation, the dataset was divided into training, validation, and testing sets in a 7:2:1 ratio. Specifically, 791 images were used for the training set, 226 images for the validation set, and the remaining 114 images for the testing set.

### 2.2 Methods

**Figure 2:**
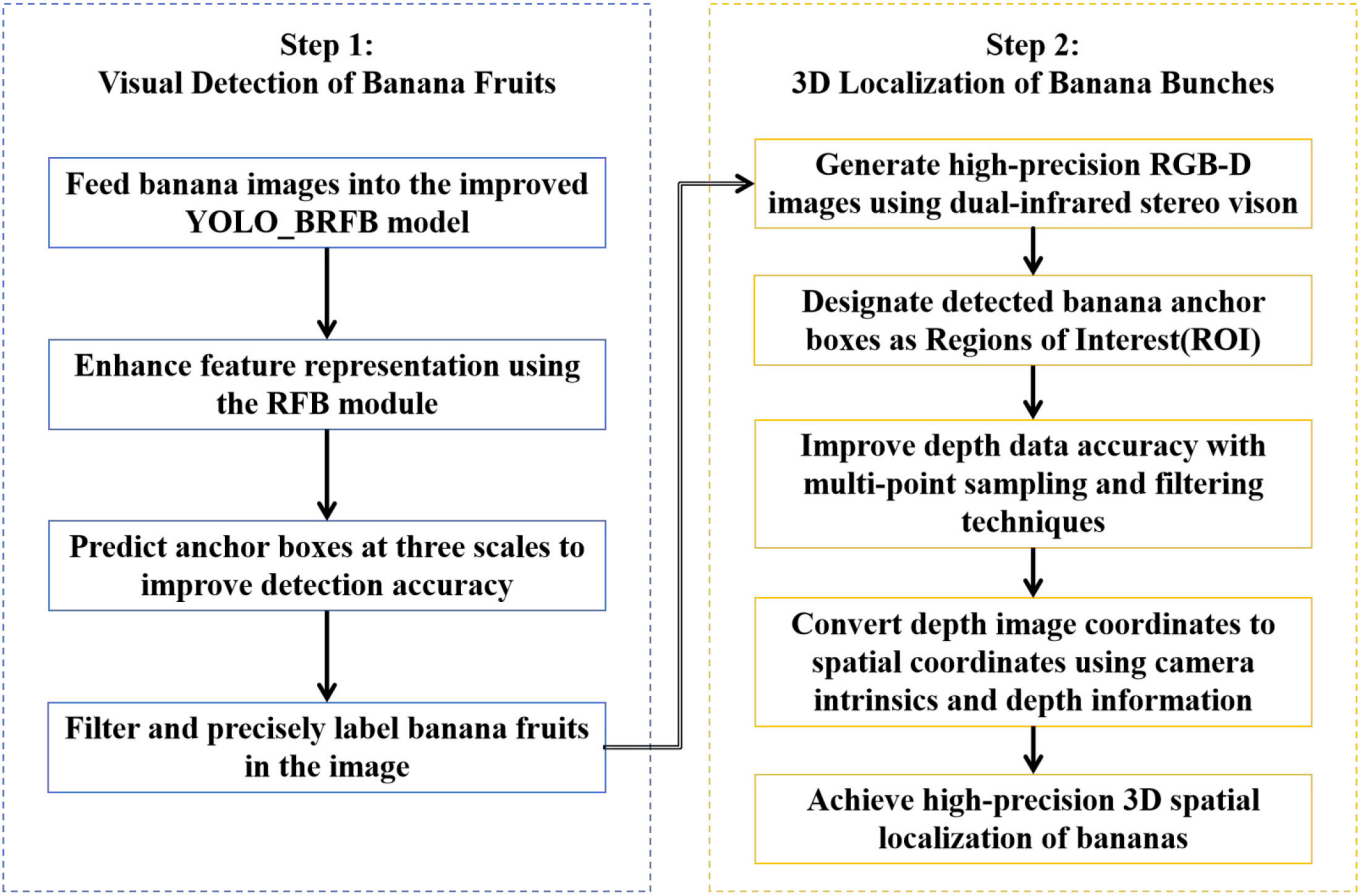
Flow chart of proposed algorithm.

This section introduces the banana bunch visual perception system, which integrates recognition and localization components into a unified framework. The system is designed to provide lightweight and real-time performance suitable for deployment on edge devices in complex agricultural environments. A detailed flowchart illustrating the overall system architecture is presented in **Figure 2**.

The framework consists of two main stages: banana bunch recognition and banana bunch localization. In the recognition stage, a lightweight YOLO-BRFB model is designed to detect banana bunches accurately. In the localization stage, a depth-based stereo vision approach is applied to determine the 3D spatial coordinates of detected banana bunches. The process begins with RGB-D image acquisition, followed by detection, and concludes with 3D coordinate extraction and refinement using filtering techniques.

#### 2.2.1 Stage 1: Banana bunch detection

Accurate identification of banana bunches in real-world orchard settings is fundamental for enabling autonomous harvesting operations. Existing fruit detection frameworks are typically categorized into two-stage models (e.g., R-CNN, Fast R-CNN, Faster R-CNN) and one-stage detectors such as the YOLO series. Unlike the two-stage pipeline that requires region proposal generation via RPN, single-stage models directly infer object locations and classes using convolutional neural networks, achieving a favorable balance between speed and accuracy.

**Figure 3:**
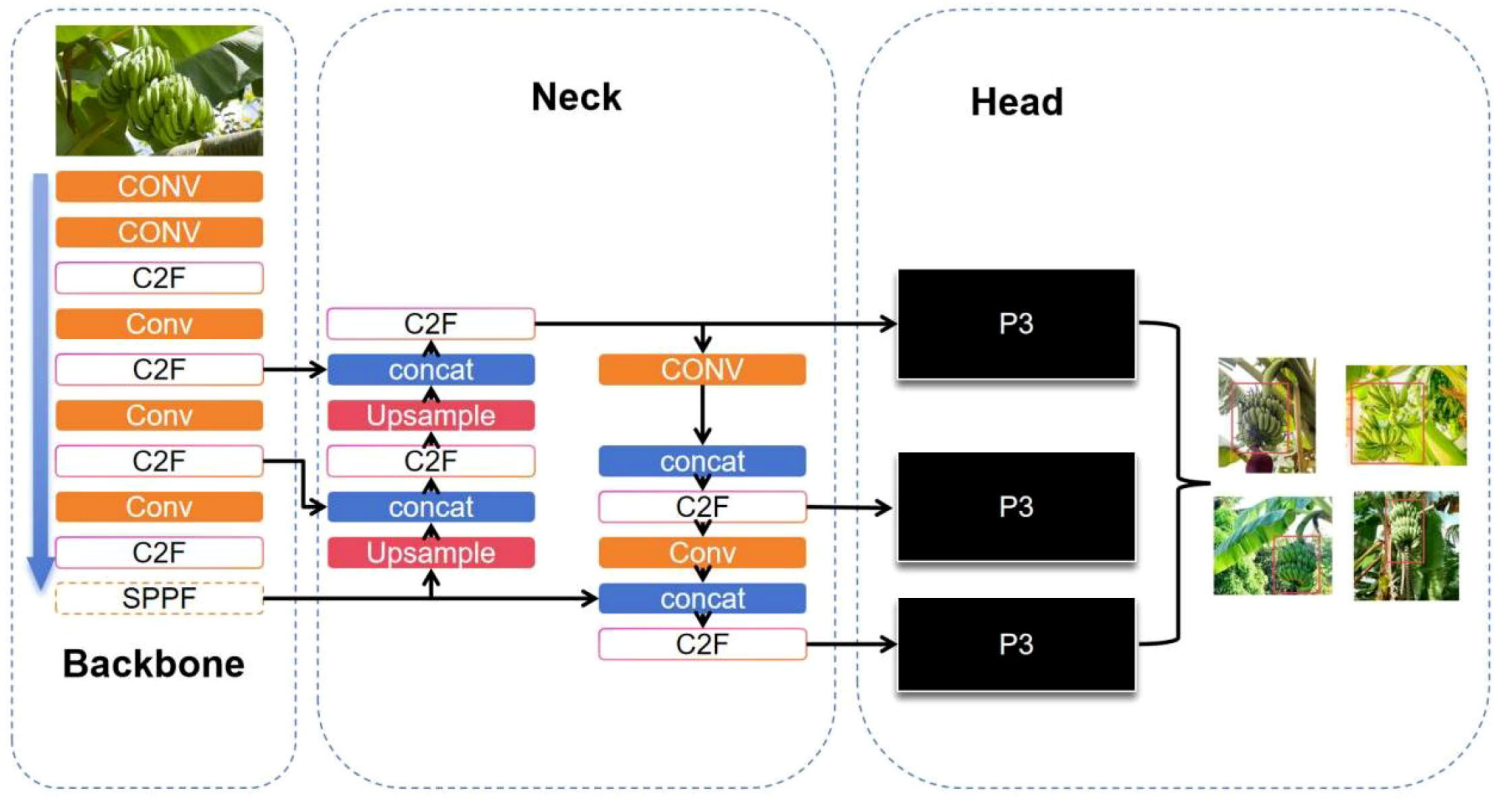
YOLOv8 network structure.

Given the relatively straightforward visual characteristics of banana bunches and the need for high real-time performance in field deployment, this study presents a streamlined detection method built upon the YOLOv8 architecture. The YOLOv8 algorithm is a state-of-the-art solution for real-time object detection, comprising four key components: input layer, backbone, neck, and head. It integrates advanced assignment and loss mechanisms, including Task Aligned Assigner, Binary Cross Entropy for classification, and a combination of DFL and CloU losses for box regression, thereby improving detection precision. The architectural design of YOLOv8 is illustrated in **Figure 3**.

The YOLOv8 model employs the Mosaic data augmentation technique at the input stage. This method enhances data diversity, enriches image backgrounds, and increases the batch size, enabling faster convergence of the training process. As a result, the efficiency of training the model is significantly improved, reducing the time required for banana recognition. Moreover, this approach facilitates seamless deployment of the model to edge GPU platforms.

The backbone network of YOLOv8 is based on an enhanced CSPDarknet architecture, incorporating three principal modules: Conv, SPPF, and the newly introduced C2F. The substitution of the original C3 block with C2F is a notable refinement that improves gradient propagation while reducing computational overhead. The model’s neck adopts a PAN-FPN hybrid topology, enabling efficient fusion of multiscale features through a combination of upsampling and downsampling pathways. This structure strengthens the network’s ability to detect objects of various sizes. In the prediction head, YOLOv8 replaces the traditional unified head with a decoupled architecture to improve detection speed without sacrificing accuracy. Furthermore, it transitions from an anchor-based mechanism to an anchor-free one, thereby expediting post-processing steps such as non-maximum suppression and boosting the model’s adaptability and robustness—critical for detecting occluded or variably sized banana bunches in complex environments.

#### 2.2.2 Stage 2: YOLO-BRFB

To further optimize the network for low-computation environments, this study replaces the SPPF module in the original YOLOv8 architecture with the BasicRFB (Receptive Field Block) module. The BasicRFB module is designed to enhance the feature representation of lightweight convolutional neural networks by simulating the varying receptive field sizes and eccentricities of the human visual system. Compared with other lightweight modules such as SimAM, CBAM, or Ghost modules, BasicRFB offers a more effective trade-off between multi-scale feature extraction capability and computational efficiency, making it particularly suitable for deployment on edge devices in complex orchard environments.

**Figure 4:**
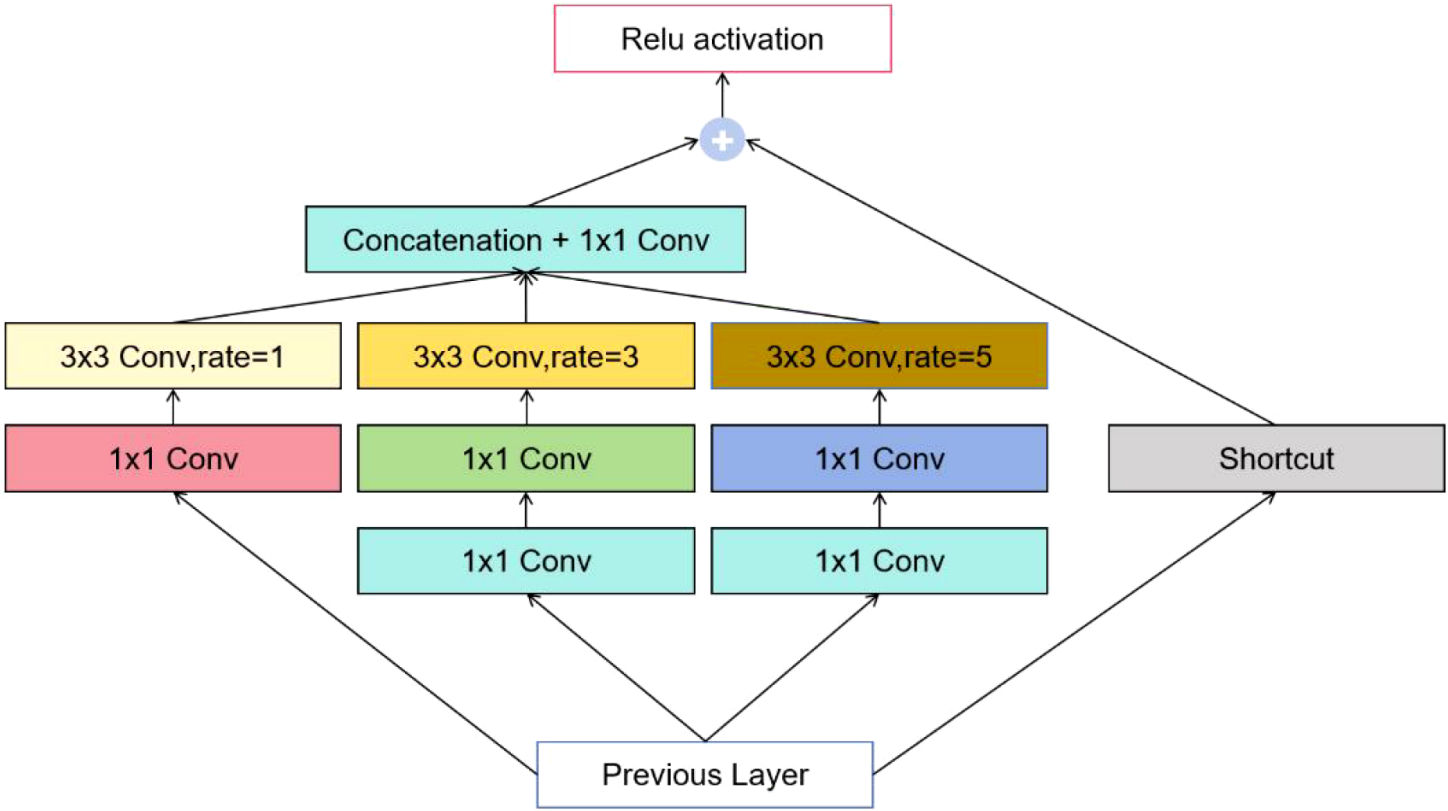
BasicRFB structure.

As illustrated in **Figure 4**, the process begins by applying three separate 1×1 convolutions to the input image for channel reduction. The resulting feature maps are then divided into three branches for processing with 1×1, 3×3, and 5×5 convolutions, respectively, to simulate receptive fields of varying sizes. For the first feature map, a 3×3 convolution with a dilation rate of 1 is used to extract feature information. For the second feature map, a 3×3 convolution is first applied to extract local information, followed by a dilated 3×3 convolution with a dilation rate of 3 to capture dispersed attention around the center. Similarly, for the third feature map, a 3×3 convolution extracts local details, which is then followed by a dilated 3×3 convolution with a dilation rate of 5 to further disperse the attention. Finally, the three feature maps are concatenated along the channel dimension, and a 1×1 convolution is applied to adjust the channel parameters. The adjusted output is then combined with the input image using a residual connection to preserve original information while enhancing feature extraction.

**Figure 5:**
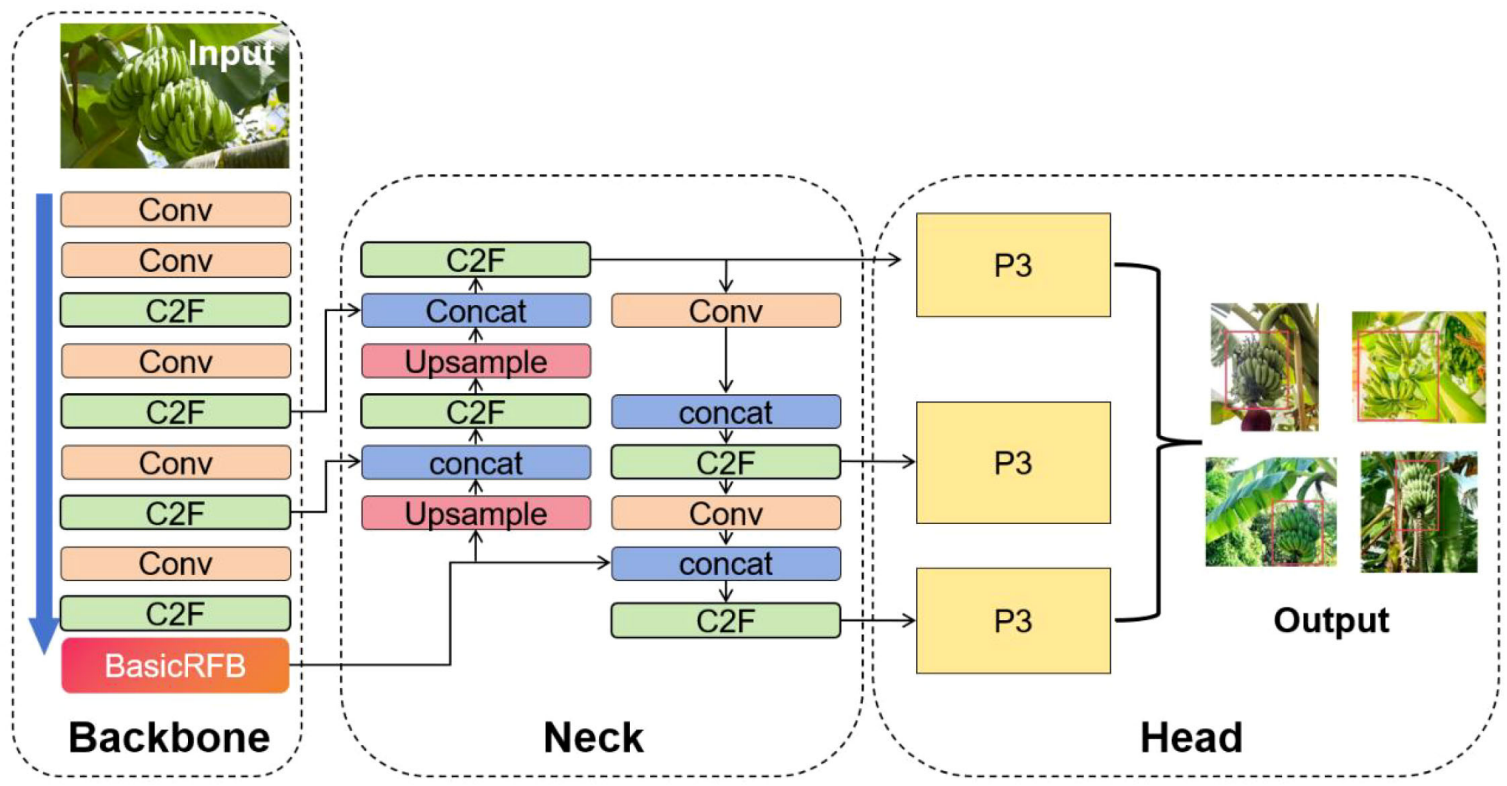
YOLO-BRFB network structure.

The model leverages a multi-branch dilated convolution structure and employs dilated convolutions to simulate eccentricity adjustments, effectively expanding the receptive field. This design enables simultaneous capture of multi-scale features, significantly enhancing model performance without increasing computational overhead. Its shallow network and narrow channel characteristics also make it suitable for deployment on low-computation-power edge devices. The overall structure of the model is shown in **Figure 5**.

#### 2.2.3 Stage 3: Banana Bunch Localization

To obtain RGB images and depth information, this study utilizes Intel’s RealSense D455 camera. Leveraging the camera’s active stereo vision technology, the 3D coordinates and depth images of banana bunches are acquired.

##### 2.2.3.1 RealSense D455

The RealSense D455 depth camera, with a measurement range of 0.2 m to 10 m, is selected for its wider and broader field of view compared to other models in the series, better suiting the requirements of banana plantation harvesting environments. The platform’s RealSense 3D camera includes a pair of left and right infrared cameras for structured light positioning, combined with an infrared dot projector for TOF positioning, as well as an RGB camera for capturing RGB images. This study specifically uses its binocular infrared feature to acquire depth images.

**Figure 6:**
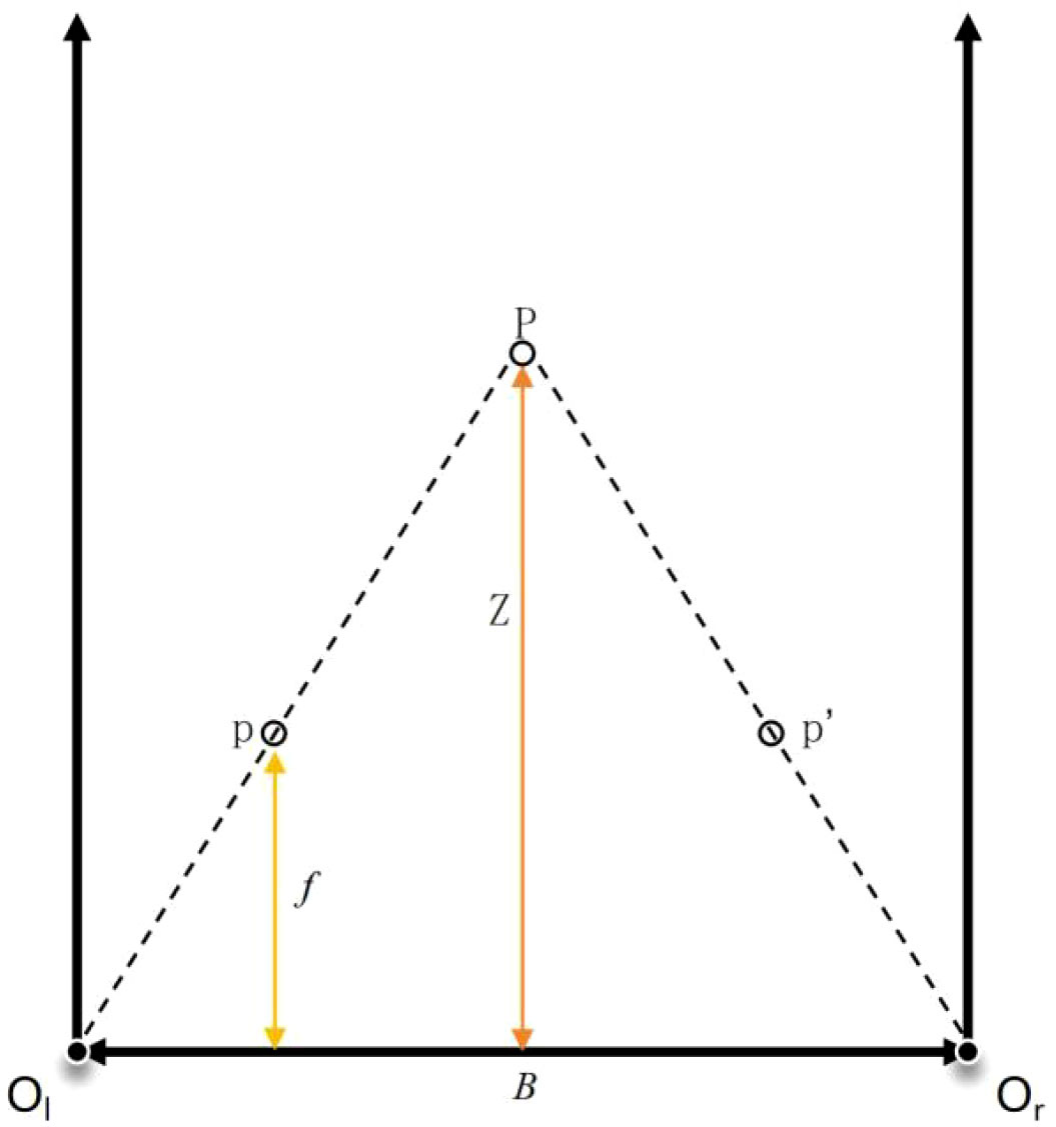
Schematic diagram of infrared active stereo vision.


As shown in **Figure 6**, the stereo system mimics the human eye by employing two identical cameras fixed at a baseline distance, with their optical axes aligned parallelly. The positions of the left and right cameras (O_l_ and O_r_), along with the pixel positions in each view (p and p), are used to determine the real-world 3D position (P) of the pixel. The disparity between the two views, caused by the horizontal displacement of the object’s position in each camera’s field of view, is calculated to derive the object’s depth. 3D coordinates based on the disparity is calculated by **Equation 1**:

(1) $$Z=\frac{f\times B}{d}$$

Where *Z* represents the distance from a point to the camera, *f* is the focal length of the camera, *B* is the baseline distance between the two cameras, and *d* is the disparity. For each pixel in the image, a 3D coordinate can be calculated using this method, thereby generating a 3D point cloud for the entire scene.

##### 2.2.3.2 Fruit localization

Since RGB images and depth images are sourced from different sensors, image alignment is necessary to ensure accurate correspondence between depth information and RGB data. This study employs the built-in SDK of the RealSense D455 camera to achieve the alignment process. This alignment utilizes the camera’s intrinsic and extrinsic parameters to map each pixel in the depth image to the corresponding pixel in the RGB image.

The alignment principle is based on the coordinate transformation between the depth camera coordinate system and the RGB camera coordinate system. Suppose the coordinates of a pixel in the depth image are ( $x_{d}$ , $y_{d}$ ) with a depth value of $Z_{d}$ . Using the intrinsic matrix $K_{d}$ of the depth camera, the 3D point (*X,Y,Z,*) in the depth camera coordinate system can be calculated by **Equation 2**:


(2) $$X=\frac{(x_{d}-c_{x}^{d})\cdot Z_{d}}{f_{x}^{d}},\;Y=\frac{(y_{d}-c_{y}^{d})\cdot Z_{d}}{f_{y}^{d}},\;Z=Z_{d}$$

Where $f_{x}^{d}$ and $f_{y}^{d}$ are the focal lengths of the depth camera, and $c_{x}^{d}$ and $c_{y}^{d}$ are the optical center positions. Then, using the extrinsic transformation matrix [R∣T], the 3D point in the depth camera coordinate system is converted to the RGB camera coordinate system by **Equation 3**:


(3) $$[\begin{array}{c} X_{r}\\ Y_{r}\\ Z_{r} \end{array}]=R\cdot [\begin{array}{c} X\\ Y\\ Z \end{array}]+T$$

Finally, using the intrinsic matrix Kr of the RGB camera, the corresponding 2D pixel coordinates ( $X_{r}$ , $Y_{r}$ ) in the RGB image can be computed as **Equation 4**:


(4) $$x_{r}=\frac{f_{x}^{r}\cdot X_{r}}{Z_{r}}+c_{x}^{r},y_{r}=\frac{f_{y}^{r}\cdot Y_{r}}{Z_{r}}+c_{y}^{r}$$

To improve the precision of 3D localization for banana bunches, this study proposes a depth value extraction strategy based on K-Means clustering. Starting with the 2D bounding box detected by the YOLO-BRFB network, the center of the bounding box is used as the sampling center, and a rectangular region is defined by scaling the bounding box size by 0.5. Within this region, 100 depth points are randomly sampled, and points with a depth value of 0 are removed to eliminate invalid data. The remaining points are then clustered using the K-Means algorithm, where the number of clusters, k, is set to 2. This step divides the points into two clusters: one representing the primary region of valid depth points and the other capturing outliers or background noise. After clustering, the cluster with the larger number of points is selected as the valid depth data. The median value of this cluster is then calculated and used as the final depth of the banana bunch. This approach minimizes the impact of noise while ensuring the depth value reflects the majority of valid points. Compared to traditional single-point methods, this K-Means-based strategy enhances accuracy and robustness by leveraging clustering to exclude spurious data points. It is computationally efficient and suitable for real-time deployment on edge devices.

## 3 Experiments and results

### 3.1 Experimental configuration and training protocol

All model evaluations were conducted under uniform conditions to ensure consistent and unbiased performance comparisons. The experimental platform consisted of a 13th Gen Intel Core i9-13900K CPU (3 GHz, 24 cores/32 threads), NVIDIA GeForce RTX 4090 GPU, Ubuntu 18.04 OS, and supporting libraries including CUDA 11.1.74, OpenCV 4.8.0, and PyTorch 2.0.1.

1. Hyperparameter Setup: Images were resized to 640×640 pixels to strike a balance between computational efficiency and visual detail. The training was executed over 500 epochs until performance metrics converged. A batch size of 24 was selected to optimize memory usage without overloading the GPU. Training commenced with a learning rate of 0.01 and a momentum factor of 0.90 to ensure fast yet stable convergence. A regularization term (weight decay = 0.0005) was applied to mitigate overfitting risks.
2. Training Enhancements: K-Means clustering was used to determine optimal anchor box dimensions. Data augmentation strategies included Mosaic to diversify spatial contexts, Mixup to enrich training variation, and horizontal flipping to enhance symmetry recognition. The use of Exponential Moving Average (EMA) smoothed parameter updates, and HSV adjustments were applied to simulate lighting variations, all contributing to improved model robustness.

### 3.2 Evaluation metrics

To comprehensively evaluate the performance of the banana detection network, several key metrics were utilized, including precision (P), recall (R), mean average precision (mAP), F1 score, inference time, and model size. These metrics are defined as follows **Equations 5**– **8**:


(5) $$P=\frac{T_{p}}{T_{p}+F_{p}}$$

(6) $$R=\frac{T_{p}}{T_{p}+F_{N}}$$

(7) $$mAP=\frac{\sum_{i=1}^{C}AP_{i}}{C}$$

(8) $$F1=\frac{2\times P\times R}{P+R}$$

Where TP represents the number of true positives correctly identified by the model, FP represents the number of false positives mistakenly classified as positive, and FN represents the number of false negatives, positive samples missed by the model. P, R, mAP, and F1 score serve as critical indicators for assessing detection performance. Additionally, inference time evaluates the speed of generating predictions for a given input, while model size reflects the storage and computational resources required. These metrics are essential for determining the effectiveness and feasibility of the model in practical applications.

### 3.3 Comparison with state-of-the-art algorithms

To verify the effectiveness of the YOLO-BRFB model, we conducted a comparative analysis against several state-of-the-art detection models, including the YOLO series (YOLOv5, YOLOv6, and YOLOv8), YOLOv8_Biformer with a bidirectional feature pyramid module, and YOLOv8_SwinTransformer, which leverages a sliding window mechanism and hierarchical structure. The evaluation was based on multiple performance metrics, including precision (P), recall (R), mean average precision (mAP), F1 score, inference time, and model size. Notably, inference time and model size were emphasized to assess the model’s suitability for real-time applications and its lightweight characteristics, both of which are essential for deployment in practical scenarios. This comparison offers a comprehensive perspective on the strengths and limitations of the YOLO-BRFB model in relation to existing advanced models.

As shown in **Table 1**, the proposed YOLO-BRFB network achieves a precision of 0.957, a recall of 0.922, an mAP of 0.961, and an F1 score of 0.939, demonstrating its superior performance in detection tasks. Notably, YOLO-BRFB achieves the highest recall and mAP among all compared models. Compared to YOLOv6, the recall and mAP are improved by 2.33% and 2.67%, respectively, while compared to YOLOv8, these metrics are further enhanced by 1.65% and 0.63%. In comparison with YOLOv8_Biformer, the proposed model achieves a higher recall and mAP by 2.9% and 0.42%, respectively, while maintaining a smaller model size and faster inference time. Although YOLOv8_Biformer achieves the highest precision of 0.97, its recall is significantly lower than YOLO-BRFB, resulting in an F1 score 0.8% lower than the proposed model. Additionally, compared to YOLOv8_Swimtransformer, YOLO-BRFB improves recall and mAP by 7.0% and 4.57%, respectively, while reducing the inference time by 33.33% and the model size by 13.78%.

**Table 1:** Comparison of detection performance of different networks.

Pattern	Precision	Recall	mAP	F1	Inference time/ms	Model_Size/MB
YOLOv5	0.965	0.897	0.942	0.929	7.4	106.8
YOLOv6	0.922	0.901	0.936	0.911	11.0	222.2
YOLOv8	0.951	0.907	0.955	0.928	7.9	87.6
YOLOv8_Biformer	0.97	0.896	0.957	0.931	9.7	88.2
YOLOv8_Swimtransformer	0.965	0.852	0.919	0.904	12.9	103.2
YOLO-BRFB	0.957	0.922	0.961	0.939	8.6	89.0

In terms of efficiency, YOLO-BRFB achieves an inference time of 8.6 ms and a model size of 89.0 MB, offering a balanced trade-off between detection accuracy and computational efficiency. Compared to YOLOv6, the inference time is reduced by 21.82%, and the model size is compressed by 59.94%. While YOLOv8 achieves a slightly faster inference time of 7.9 ms, YOLO-BRFB reduces the model size by 12.97%. In comparison with YOLOv8_Biformer, the inference time is reduced by 11.34% with a 1.02% smaller model size, demonstrating superior real-time performance. Although YOLOv8_Swimtransformer shows competitive performance in precision, its recall and mAP are significantly lower than YOLO-BRFB, and its model size and inference time increase substantially by 15.96% and 50.0%, respectively. These results indicate that YOLO-BRFB effectively balances detection accuracy, computational cost, and model compactness, making it highly suitable for real-time detection tasks.

YOLO-BRFB demonstrates an optimal combination of accuracy and efficiency, outperforming other state-of-the-art models in both detection precision and practical feasibility. This advantage stems from the integration of the BasicRFB module, which replaces standard convolution layers with dynamically adjustable kernels, enhancing the model’s ability to capture intricate data features. Additionally, the use of a multi-branch dilated convolution structure expands the receptive field by simulating eccentricity adjustments, enabling efficient multi-scale feature extraction. These design innovations not only maintain high detection accuracy but also reduce computational and structural complexity, making YOLO-BRFB particularly suited for deployment in resource-constrained environments. In the context of banana harvesting robots, this ensures accurate fruit detection and localization while meeting the real-time processing demands of edge devices, thereby enhancing operational efficiency and reliability in practical applications.

**Figure 7:**
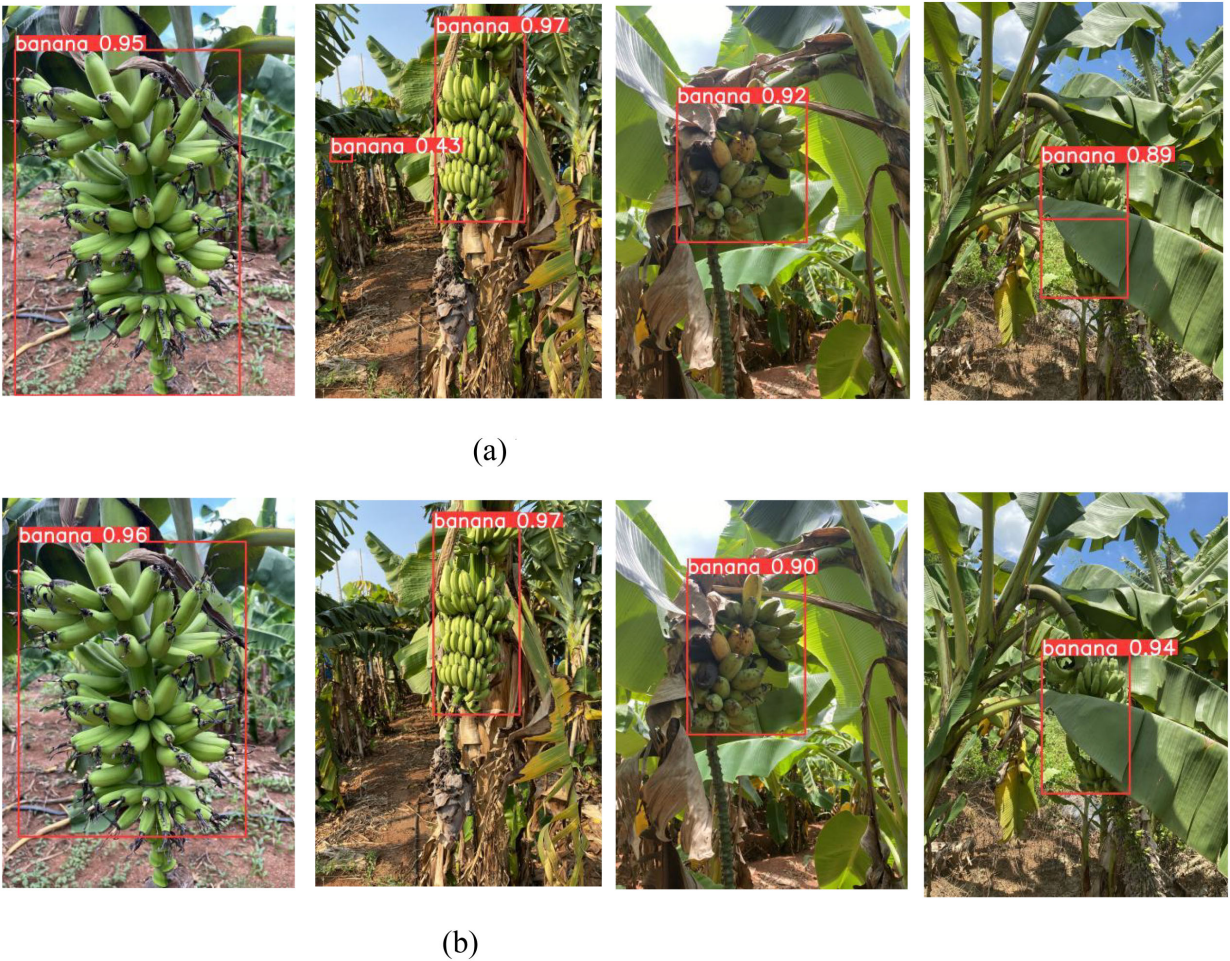
Comparison before and after YOLOv8 network improvement. **(a)** YOLOv8 network **(b)** YOLO-BRFB network.

**Figure 7** presents a comparative evaluation of the proposed YOLO-BRFB model against the baseline YOLOv8 under challenging conditions such as occlusion and suboptimal lighting. The enhanced YOLO-BRFB network consistently outperforms its counterpart, offering higher detection accuracy and more precise localization of banana bunches. In occluded scenes, YOLOv8 tends to produce redundant or misaligned bounding boxes, negatively impacting detection reliability. Conversely, YOLO-BRFB effectively mitigates these issues through refined feature extraction and improved spatial awareness, leading to a significant reduction in false positives and missed targets. Furthermore, under adverse lighting scenarios-including high contrast, shadowing, and dim illumination-YOLOv8 exhibits a marked decline in performance, whereas YOLO-BRFB maintains stable and accurate predictions. These results confirm that the proposed model demonstrates superior adaptability and resilience, making it particularly suitable for deployment in dynamic agricultural environments such as automated banana harvesting.

### 3.4 Field experiments

#### 3.4.1 Deployment on edge computing devices

In practical orchard environments, limitations in computational resources and inconsistent network connectivity pose significant challenges to achieving low-latency fruit recognition. To address these issues, this study deploys the proposed YOLO-BRFB model on an Nvidia Orin NX edge computing device, enabling end-to-end detection and localization of banana bunches without reliance on cloud processing. This deployment enhances the system’s ability to deliver accurate and stable results under resource-constrained conditions. A comparative evaluation was conducted before and after deployment to assess performance across diverse real-world orchard scenarios.

As shown in **Table 2**, the YOLO-BRFB model exhibits consistent detection performance across two devices, Nvidia A6000 and Nvidia Orin NX, achieving a Precision (P) of 0.957, Recall (R) of 0.922, and mAP of 0.961. This consistency underscores the model’s robustness and adaptability in varying computational environments. In terms of inference time, YOLO-BRFB achieves 8.60 ms on the A6000 and 109.80 ms on the Orin NX. Although the Orin NX exhibits longer processing times due to its lower computational capacity, the inference time still meets real-time processing requirements. This demonstrates that the proposed solution achieves satisfactory real-time performance even on resource-constrained devices, offering a cost-effective alternative without compromising operational efficiency. It is important to note that the reported inference time reflects only the model forward pass and excludes preprocessing and postprocessing operations. The memory usage further highlights the advantages of the model and device. On the Orin NX, YOLO-BRFB requires only 1.700 GB of GPU memory compared to 1.975 GB on the A6000. This improvement not only reflects the optimized design of the model, including the efficient use of computational resources and reduced structural complexity, but also the advanced inference efficiency of the Orin NX hardware itself. Together, these factors enable deployment on devices with limited memory and computational capabilities. Experimental results confirm that YOLO-BRFB provides a practical solution for edge computing scenarios, especially in the context of banana harvesting robots. The model balances accuracy, efficiency, and cost-effectiveness, making it well-suited for field applications requiring compact, low-power, and reliable performance under resource constraints.

**Table 2:** Performance comparison of YOLO-BRFB on A6000 and Nvidia Orin NX.

Device	Precision (P)	Recall (R)	mAP	Inference time (ms)	GPU memory usage (GB)
A6000	0.957	0.922	0.961	8.60	1.975
Nvidia Orin NX	0.957	0.922	0.961	109.80	1.700

#### 3.4.2 Performance evaluation under different scenarios

Given the morphological structure of banana bunches, frequent overlap between leaves and fruits is common in natural orchard settings. In addition, varying illumination conditions—such as strong sunlight, shading, and backlighting—further complicate visual detection tasks. To comprehensively assess the robustness of the proposed model, practical scenes were divided into four representative categories: occlusion, clear visibility, backlit, and high-exposure lighting. This classification enables a detailed evaluation of the model’s adaptability under real-world environmental complexity.

As shown in **Table 3**, under non-occlusion conditions, the YOLO-BRFB model achieves outstanding results, with precision, recall, mAP, and F1-score of 0.995, 0.960, 0.991, and 0.977, respectively. These values highlight the model’s ability to perform optimally when fruits are fully visible. In occlusion scenarios, the precision decreases by 7.0% to 0.925, and the recall drops by 3.1% to 0.930. Despite these reductions, the model still maintains a high mAP of 0.966 and an F1-score of 0.927, demonstrating its capacity to detect partially obscured fruits effectively. For direct lighting conditions, the precision, recall, mAP, and F1-score reach 0.991, 0.965, 0.989, and 0.978, respectively, with minimal changes compared to non-occlusion scenarios, showing strong robustness against high-intensity lighting. Under backlighting conditions, the performance decreases slightly, with precision and recall dropping to 0.921 and 0.905, respectively. This results in an mAP of 0.967 and an F1-score of 0.913, reflecting the model’s adaptability to challenging lighting environments, though with a marginal impact on detection accuracy. These results highlight the YOLO-BRFB model’s ability to adapt to diverse real-world conditions, ensuring reliable and accurate fruit detection in scenarios commonly encountered in banana orchards.

**Table 3:** Comparison of model performance under different scenarios.

Scenes	Precision	Recall	mAP	F1
Occlusion	0.925	0.93	0.966	0.927
Non-occlusion	0.995	0.96	0.991	0.977
Backlighting	0.921	0.905	0.967	0.913
Direct lighting	0.991	0.965	0.989	0.978

**Figure 8:**
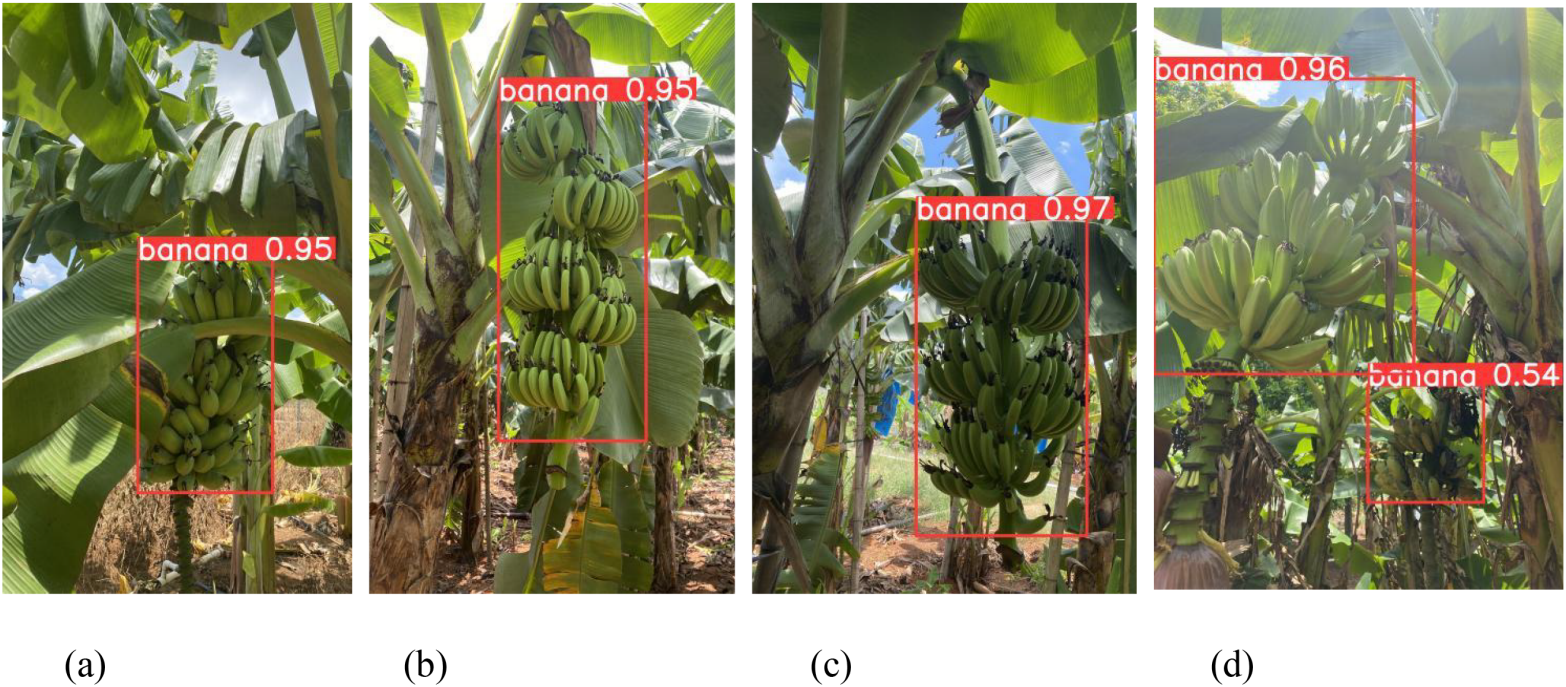
Recognition renderings in different scenarios. **(a)** Occlusion **(b)** Non-occlusion **(c)** Backlighting **(d)** Direct lighting.

As illustrated in **Figure 8**, the YOLO-BRFB model delivers reliable detection outcomes across multiple environmental settings, effectively identifying banana bunches under diverse visual conditions.

#### 3.4.3 Three-dimensional banana bunch localization

**Figure 9:**
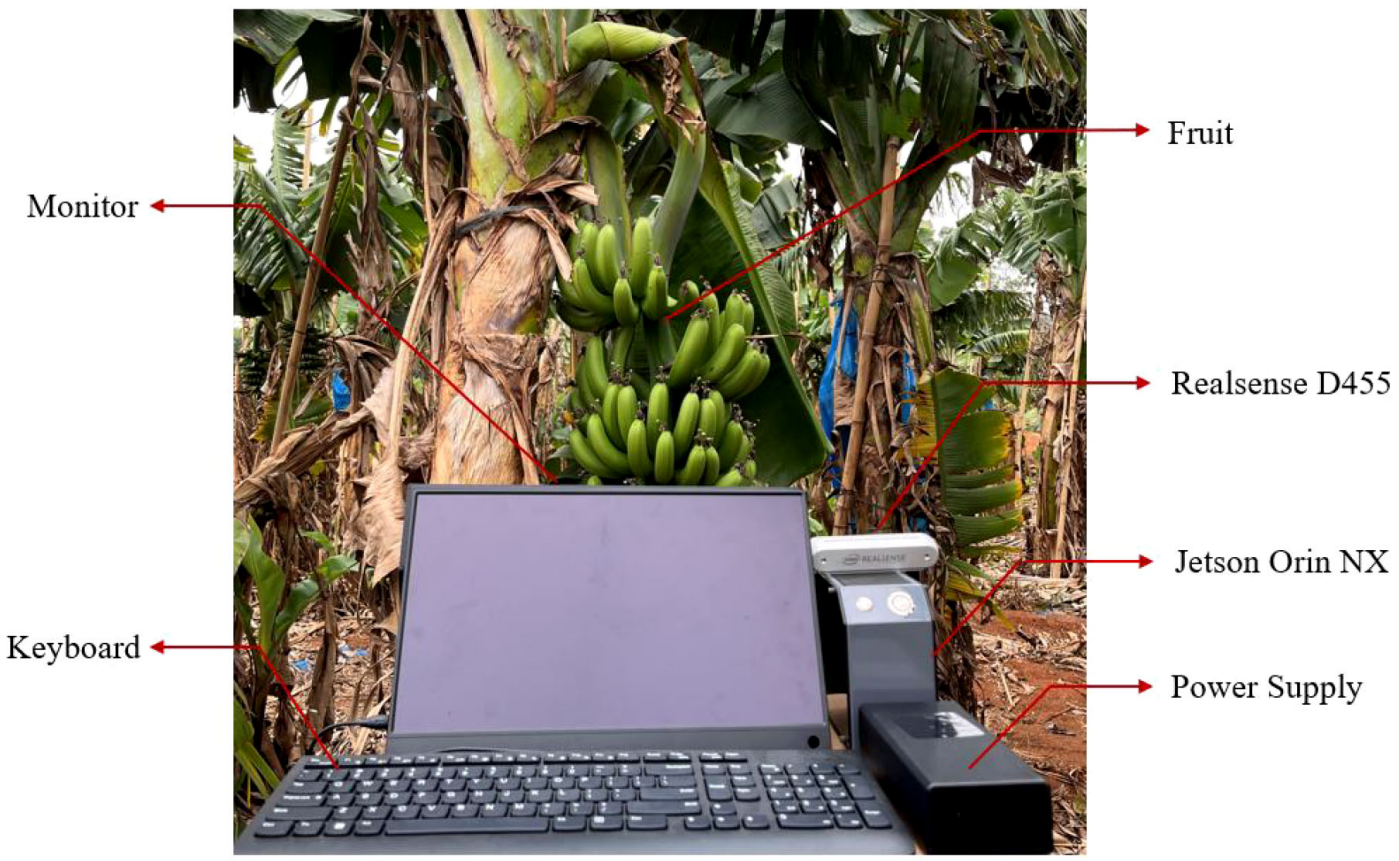
Working scene diagram of the banana bunch visual perception system.

An experimental protocol was developed to validate the system’s localization accuracy under actual deployment scenarios. As shown in **Figure 9**, the setup included a Realsense D455 depth camera, a Jetson Orin NX module for on-device processing, a display unit for visual feedback, and a laser rangefinder to establish ground-truth coordinates. The depth sensor, mounted on a stable tripod, acquired real-time depth images, which were processed by the YOLOv8-BRFB network on the edge device to compute 3D spatial coordinates. The true positions of the banana bunches were independently measured using the rangefinder, and these values were compared against the predicted outputs. Metrics such as positional error and localization accuracy were recorded to assess the reliability of the 3D positioning pipeline.

As shown in **Table 4**, it was observed that when the distance between the sensor and the fruit was less than 0.3 meters, the fruit was within the blind spot of the depth camera, making it impossible to obtain depth data, as seen in the first and second trials where no 3D coordinates were detected. When the distance exceeded 1.2 meters, the error significantly increased, resulting in inaccurate positioning, as reflected in the data from the 10th and 11th trials. This indicates that the effective working range for accurate positioning is between 0.3 and 1.2 meters. Excluding the first and second trials, the data from **Table 4** shows that the coordinate errors are presented in millimeters (mm) and are listed in the format (X, Y, Z), corresponding respectively to the horizontal (X-axis), vertical (Y-axis), and depth (Z-axis) deviations between predicted and actual values. Specifically, the maximum error along the X-axis was 33 mm, while the minimum was 4 mm, with an average error of 12.33 mm. For the Y-axis, the maximum error was 35 mm, the minimum was 1 mm, and the average error was 11.11 mm. For the Z-axis, the maximum error was 46 mm, the minimum was 4 mm, and the average error was 16.33 mm. Although some errors were observed, they remain within an acceptable range for practical applications.

**Table 4:** 3D Coordinate comparison and errors for banana bunch localization.

No.	Real 3D coordinates (m)	Detection 3D coordinates (m)	Coordinate error (mm)
1	(0.113, 0.143, 0.086)	(0.000, 0.000, 0.000)	/
2	(-0.125, 0.146, 0.135)	(0.000, 0.000, 0.000)	/
3	(-0.097, 0.055, 0.325)	(-0.102, 0.042, 0.331)	(5, 13, 6)
4	(-0.044, 0.080, 0.459)	(-0.058, 0.071, 0.463)	(14, 9, 4)
5	(0.106, 0.051, 0.575)	(0,101, 0.064, 0.581)	(5, 13, 6)
6	(0.067, 0.145, 0.671)	(0.059, 0.152, 0.688)	(8, 7, 17)
7	(0.098, 0.239, 0.751)	(0.094, 0.229, 0.760)	(4, 10, 9)
8	(-0.127, 0.370, 0.829)	(-0.134, 0.369, 0.835)	(7, 1, 6)
9	(0.327, 0.338, 0.967)	(0.341, 0.342, 0.987)	(14, 4, 20)
10	(0.144, 0.346, 0.1199)	(0.165, 0.354, 0.1238)	(21, 8, 39)
11	(-0.167, 0.289, 0.1293)	(-0.134, 0.324, 0.1339)	(33, 35, 46)

**Figure 10:**
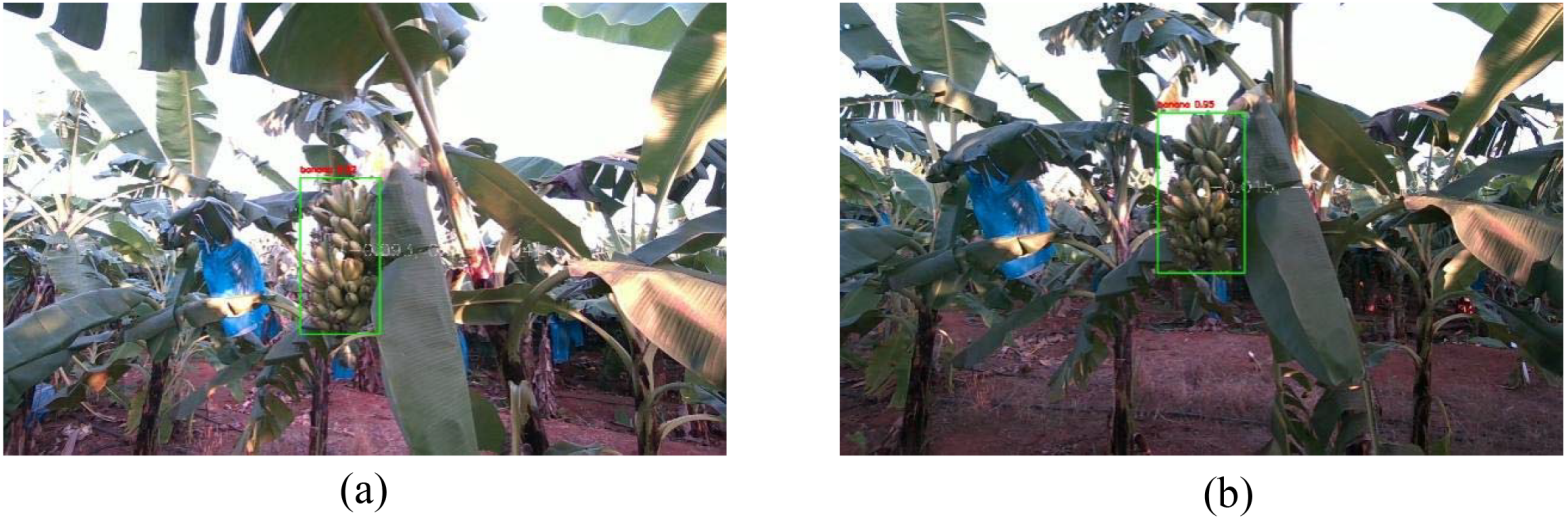
System working effect diagram in complex orchard scenes. **(a)** Occlusion and direct light scenes **(b)** Non-occlusion and Backlighting scenes.

Localization discrepancies can be attributed to several factors, such as sensor resolution limitations, the irregular geometry of banana clusters, and variations in ambient lighting. Despite these challenges, the system consistently achieved accurate positioning within the defined operational range. Moreover, as depicted in **Figure 10**, the detection and localization framework maintained reliable performance under diverse conditions, including occlusion and variable lighting, reinforcing its suitability for real-world applications.

## 4 Discussion

This research presents a lightweight and efficient banana bunch detection and localization framework, YOLO-BRFB, specifically designed to overcome the challenges posed by complex lighting and occlusion in orchard environments. By integrating the BasicRFB module into the YOLOv8 architecture, the model achieves a favorable trade-off between detection accuracy and computational cost. The system was successfully implemented on the Nvidia Orin NX edge device, where it demonstrated strong real-time performance and low resource consumption.

Additionally, we incorporated binocular active vision to enhance 3D localization, achieving high spatial accuracy validated through field experiments. Compared to earlier studies—such as those by Duan et al. (23), Cai et al. (25), and Wang et al. (27) - that utilized YOLO-based networks for fruit detection, our approach significantly reduces model complexity while enabling edge-side deployment. Prior 3D localization attempts, including Zhou et al. (28) with structured light and Yao et al. (36) with TOF sensors, have been constrained by environmental sensitivity and distance limitations. In contrast, our binocular infrared vision method offers robust depth perception across a wider range of conditions.

The core contribution of this work lies in combining lightweight object detection with practical edge deployment and robust 3D localization under real orchard constraints. Nonetheless, some limitations remain: performance degradation under extreme lighting—especially backlighting—and slight latency increases under high-throughput conditions. Future improvements will focus on optimizing model inference efficiency, enhancing low-light robustness, and integrating complementary sensing technologies such as LiDAR or multispectral imaging to further improve adaptability across diverse agricultural settings.

## 5 Conclusions

In this study, we proposed a lightweight and efficient banana bunches detection and localization system, YOLO-BRFB, designed to address the challenges of complex lighting conditions in banana orchards. By integrating the BasicRFB module into the YOLOv8, YOLO-BRFB achieves a balance between high accuracy and model compactness. Experimental results demonstrated its superiority, achieving a precision of 0.957, a recall of 0.922, an mAP of 0.961, and an F1-score of 0.939, outperforming state-of-the-art models such as YOLOv6 and YOLOv8. Notably, compared to YOLOv6, YOLO-BRFB improves recall and mAP by 2.33% and 2.67%, respectively, while reducing inference time by 21.82% and model size by 59.94%.

The deployment on the Nvidia Orin NX edge device further validates its practicality, achieving an inference time of 109.80 ms and requiring only 1.700 GB of GPU memory, which is 14% lower than on the Nvidia A6000. Despite the lower computational capacity of the Orin NX, YOLO-BRFB maintains real-time processing capabilities, highlighting its suitability for resource-constrained environments. Additionally, the binocular active vision module enables precise 3D localization of banana bunches, with an effective range of 0.3–1.2 meters. Within this range, the average localization error was 12.33 mm along the X-axis, 11.11 mm along the Y-axis, and 16.33 mm along the Z-axis, meeting the accuracy requirements for practical applications.

The system demonstrates robust detection performance under diverse orchard conditions. For non-occlusion scenarios, it achieves a precision of 0.995 and an mAP of 0.991. Under occlusion, backlighting, and direct lighting conditions, it maintains high accuracy, with mAP values of 0.966, 0.967, and 0.989, respectively. These results underscore the model’s robustness and adaptability to varying environmental factors, ensuring reliable detection in real-world orchards. By balancing performance and cost-effectiveness, YOLO-BRFB provides a practical solution for banana harvesting robots.

## Data Availability

The raw data supporting the conclusions of this article will be made available by the authors, without undue reservation.
